# Examining the Prey Mass of Terrestrial and Aquatic Carnivorous Mammals: Minimum, Maximum and Range

**DOI:** 10.1371/journal.pone.0106402

**Published:** 2014-08-27

**Authors:** Marlee A. Tucker, Tracey L. Rogers

**Affiliations:** Evolution and Ecology Research Centre, School of Biological, Earth and Environmental Sciences, The University of New South Wales, Sydney, New South Wales, Australia; Max Planck Institute for Evolutionary Anthropology, Germany

## Abstract

Predator-prey body mass relationships are a vital part of food webs across ecosystems and provide key information for predicting the susceptibility of carnivore populations to extinction. Despite this, there has been limited research on the minimum and maximum prey size of mammalian carnivores. Without information on large-scale patterns of prey mass, we limit our understanding of predation pressure, trophic cascades and susceptibility of carnivores to decreasing prey populations. The majority of studies that examine predator-prey body mass relationships focus on either a single or a subset of mammalian species, which limits the strength of our models as well as their broader application. We examine the relationship between predator body mass and the minimum, maximum and range of their prey's body mass across 108 mammalian carnivores, from weasels to baleen whales (Carnivora and Cetacea). We test whether mammals show a positive relationship between prey and predator body mass, as in reptiles and birds, as well as examine how environment (aquatic and terrestrial) and phylogenetic relatedness play a role in this relationship. We found that phylogenetic relatedness is a strong driver of predator-prey mass patterns in carnivorous mammals and accounts for a higher proportion of variance compared with the biological drivers of body mass and environment. We show a positive predator-prey body mass pattern for terrestrial mammals as found in reptiles and birds, but no relationship for aquatic mammals. Our results will benefit our understanding of trophic interactions, the susceptibility of carnivores to population declines and the role of carnivores within ecosystems.

## Introduction

Examining patterns in predator-prey relationships provides information on predation pressure (e.g. on specific size guilds) [1], [2], the impact of decreasing prey species on predators [3] and the potential for trophic cascades and the collapse of prey populations [4]–[6]. However, previous research on predator-prey body mass relationships in mammalian carnivores has focused upon the mean mass of prey, largely ignoring the minimum and maximum body mass of prey consumed by predators. It is important to include the minimum, maximum and range of prey mass consumed as it allows the examination of the upper and lower limits of carnivore prey selection. In addition, prey selection provides information such as energetic requirements (e.g. intake rates), which is often used for predicting the susceptibility of carnivores to population declines, the role of carnivores within ecosystems and community structure [13].

Larger-sized predators can utilise a wide variety of prey types because they have large home ranges [7] that provide access to a diversity prey species [8], as well as a wide gape size that allows them to feed on prey of a variety of sizes. Despite this, large predators tend to eat larger-sized prey [13]. It is not always profitable for large species to feed on small-sized prey due to capture inefficiency as it is costly to pursue small-sized prey in relation to the small energetic benefit gained [9]. The minimum and maximum size of prey should scale positively with predator body mass, resulting in there being no relationship between predator body mass and diversity of prey size (i.e. dietary niche breadth - DNB) [10], [11]. However, if maximum prey size scales positively with predator mass and minimum prey size does not this will result in a larger diversity of prey size for larger predators (i.e. wider DNB).

Our knowledge of mammalian broad-scale patterns of prey-size range is limited. There has been limited work investigating the prey mass of African predators and its effect on the system [12]. However, this work largely focuses upon predation pressure on prey species, particularly herbivorous mammals. The remaining predator-prey body mass research is based the mean prey mass of predators [13], [14]. Investigations into other animal groups include predatory fish [15], [16], reptiles [11], [17] and birds [10], where there is a general consensus that there is a positive relationship between predator body mass and prey minimum, maximum and range in mass, except for fish where the evidence is conflicting (positive or no relationship between predator mass and minimum prey mass).

Using minimum, maximum and range of prey mass for 108 carnivorous mammals from the orders Carnivora and Cetacea, we investigated the nature of the relationship between carnivore body mass and prey body mass and how living in either the marine or terrestrial environment has impacted this relationship. This study has two objectives: first to examine the influence of physical environment on minimum, maximum and range of prey mass; and second to investigate the influence physical environment has had on the distribution of minimum, maximum and range of prey mass. Based on previous research [11], [13], we predict that prey mass (minimum, maximum and range) will be positively correlated with predator body mass for terrestrial carnivores. However, for marine carnivores there could be two possible outcomes: first, prey mass (minimum, maximum and range) could be positively correlated with body mass similar to terrestrial carnivores and other marine non-mammalian predators [16]; or second, there could be no relationship between prey mass (minimum, maximum and range) and predator body mass. No relationship between predator mass and prey mass is a possibility due to the high abundance of small species that form dense aggregations in aquatic environments (e.g. krill or fish), which lead to an increase in the encounter rates between aquatic predators and these small prey species. With both small and large predators exposed to these abundant food resources, this would result in both small and large predators feeding upon small prey species and therefore suggest no relationship between predator mass and prey mass in aquatic systems.

By examining the moments (e.g. mean, mode, skewness etc.) of the prey mass distributions, we can gather information on how the mass of the prey consumed by carnivorous mammals differs or is similar across different environments. This information is important for building our knowledge of predator-prey relationships and the drivers behind these relationships.

## Materials and Methods

### Ethics statement

All data in this study were extracted from published sources; hence no permission or approval for obtaining the data was required.

### Database

Data were collated on the minimum and maximum prey mass (kg) consumed by 108 carnivorous mammal species (Appendix S1). Table 1 provides a summary of the orders and families sampled. Prey mass range was calculated by subtracting the minimum prey mass from the maximum prey mass. Mean body mass (kg) was also collected for these 108 predator species using the database PanTHERIA [18]. All carnivores were classified as terrestrial or aquatic, where aquatic species forage in water to survive (e.g. foraging) and terrestrial species forage on land to survive. All values including carnivore mass and prey mass were log_10_ transformed prior to all analyses.

**Table 1:** Summary of the orders and families included in our study sample.

Order	Family
Carnivora	Canidae
	Felidae
	Herpestidae
	Hyaenidae
	Mephitidae
	Mustelidae
	Otariidae
	Phocidae
	Procyonidae
	Ursidae
	Viverridae
Cetacea	Balaenidae
	Balaenopteridae
	Cetotheriidae
	Delphindae
	Delphinidae
	Eschrichtiidae
	Kogiidae
	Monodontidae
	Phocoenidae
	Physeteridae
	Pontoporiidae
	Ziphiidae

### Phylogeny

We required a single phylogenetic tree to examine minimum prey mass, maximum prey mass and prey mass range in carnivorous mammals. Phylogenetic information was obtained from the Fritz *et al*. [19] mammal supertree containing 5,020 species and branch lengths proportional to time since divergence. This tree was pruned using Mesquite ver 2.74 [20] to create the tree (n = 108) based on the data in our database. *Sotalia guianensis* (Guiana dolphin) was positioned within the pruned tree based on the topologies of Caballero *et al*. [21]. Due to insufficient phylogenetic information, the Fritz *et al*. [19] tree included soft polytomies where more than two species diverge at a single point in time. To resolve the polytomies, we used a semi-automated polytomy resolver for dated phylogenies [22]. The polytomy resolution involved two steps; 1) R 3.0.2 [23] was used to create an XML input file containing topology constraints and input commands for BEAST, and 2) the XML input file was run through the program BEAST 1.8 [24] which uses a Bayesian Markov chain Monte Carlo (MCMC) algorithm to permute the unresolved relationships within the tree based on the birth-death model. This produced 1,000 alternative phylogenetic trees to be used for the phylogenetic comparative analyses and the ancestral state reconstructions.

### Analyses

We applied a model selection approach to test the level of support for alternative models of prey mass evolution. The best model was selected using second-order Akaike's information criterion with a correction for sample size (AICc; [25]). The model with the lowest AICc value reflects the model with the highest support, although any other models within two units of the lowest model were also considered to be likely candidates. We selected the cut-off of <2.0 ΔAIC based on previous studies who have identified that models below this threshold are generally equally supported, models between 4–7 ΔAIC have some support and models >10 ΔAIC have no support [26]–[28]. To compute AICc values, we applied each model as a phylogenetic generalized least squares (PGLS) regression using the CAPER package in R [29] to each of the 1000 trees (see previous section). PGLS regression also computes a λ parameter using maximum likelihood that estimates whether the extent of phenotypic variation among species (e.g., mean body mass and associated home range size) is correlated to phylogeny. When λ is close to 1, phenotypic differentiation among present-day taxa reflects the phylogenetic relationships among those species and is the product of Brownian evolution. When λ is close to 0 phenotypic differentiation is unrelated to phylogeny and might be the outcome of adaptive evolution [30].

We performed three separate PGLS analyses for minimum prey mass, maximum prey mass and range of prey mass respectively. For each we examined the level of support of the relationship between prey mass (minimum, maximum or range), carnivore body mass and environment across 108 species. The models were formulated as; (a) *β*_0_+*β*_mass_* *β*_environment_, where environment was coded as “terrestrial” or “aquatic” and included an interaction term between carnivore mass and environment; (b) *β*_0_+*β*_mass_, which assumed body mass was the only variable predicting prey mass; (c) *β*_0_, the evolutionary null model in which no predictor variable was included and subsequently modelled variance in species prey mass as the outcome of Brownian evolution (i.e. under Brownian motion, trait evolution proceeds as a random walk through trait space and Brownian motion has been proposed as a null model of evolution for testing hypotheses of trait evolution [31]).

To examine the effect of phylogeny and ecology on the minimum and maximum prey mass of carnivores, we ran variance component analyses [32]. Variance was examined between species, focusing on the contribution of order, family, genus, mass and environment (aquatic or terrestrial). Variance components analysis was performed using the lme4 package [33] in R version 2.13.2.

To gain an understanding on the shape of the prey mass distributions across species and environments, we extracted the descriptive statistics including the mean, median, mode, range, minimum, maximum, standard deviation (S.D.), skewness and kurtosis. Skewness measures the degree of asymmetry of a distribution. If the skewness value is positive the data has a right skewed distribution and a negative value suggests left skewed data. Kurtosis measures height of the curve relative to its standard deviations. Data with a peaked distribution with values around zero (i.e. normal distribution) have a positive kurtosis value, whereas negative values between 0 and -1 implies that the data has a flat distribution and values lower than −1.5 suggest a bimodal distribution.

## Results

### Phylogenetic Generalized Least Squares Regression

The model including an interaction between body mass and environment (mass-environment) was the best supported model for minimum prey mass, maximum prey mass and prey mass range (Table 2). However, for the maximum prey mass and prey mass range there was less than 2 ΔAIC units between the mass model and the mass-environment model, suggesting that these models are equally supported. In both cases, the addition of environment explained a limited amount of additional variance (3% for both maximum prey and prey range) however for minimum prey mass, environment explained an additional 7% of variance. The phylogenetic signal (λ) was consistently high for the mass model for minimum, maximum and prey size range (>0.7). Lambda however, was mixed for the mass-environment models, where it was low (<0.5) for the maximum prey and prey range size, it was higher (0.62) for the minimum prey mass.

**Table 2:** Level of support for explanatory models of prey mass evolution in carnivorous mammals.

Prey mass	Model	ΔAICc	ΔAICc 95% CI (upper, lower)	Lambda	Effect size (r)
Minimum	*β*_0_+*β*_mass_ x *β*_environment_	0.0	NA	0.62	0.28
	*β*_0_+*β*_mass_	5.8	4.99, 6.58	0.71	0.10
	*β*_0_	5.5	4.78, 6.28	0.64	NA
Maximum	*β*_0_+*β*_mass_ x *β*_environment_	0.0	NA	0.48	0.28
	*β*_0_+*β*_mass_	0.9	0.04, 2.07	0.74	0.23
	*β*_0_	4.8	4.23, 5.29	0.57	NA
Range	*β*_0_+*β*_mass_ x *β*_environment_	0.0	NA	0.30	0.28
	*β*_0_+*β*_mass_	1.6	0.23, 3.1	0.73	0.23
	*β*_0_	5.3	4.54, 5.84	0.57	NA

Results are from phylogenetic least squares (PGLS) regression analyses computed for 1000 alternative resolutions of the mammalian phylogeny. Model terms include carnivore body mass (*β*_mass_), environment either aquatic or terrestrial (*β*_environment_) and the intercept (*β*_0_).

**Figure 1:**
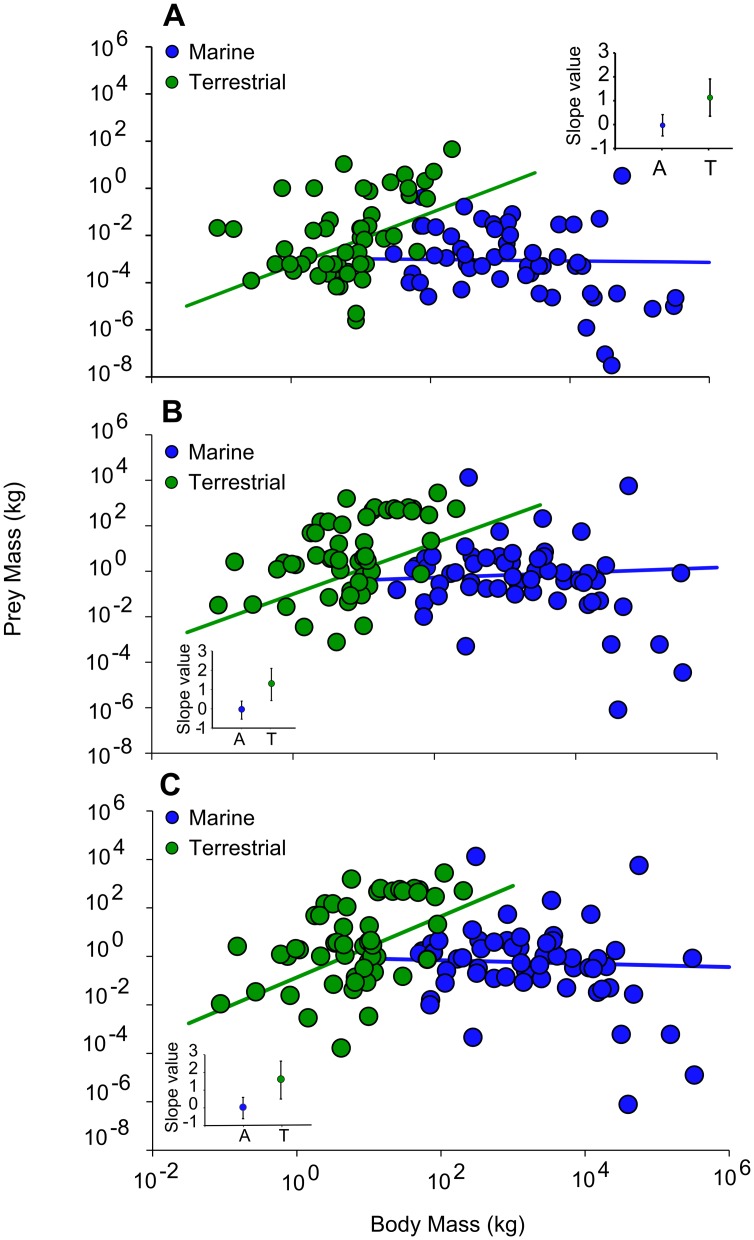
Minimum prey mass (A), maximum prey mass (B) and prey mass range (C) as a function of carnivore body mass compared for terrestrial (green circles) and aquatic (blue circles) species. Each datum represents a species mean value. The solid green line is the phylogenetic regression of terrestrial mammals: (A) log(Y) = 1.13log(X)-3.3, (B) log(Y) = 1.12log(X)-1.01 and (C) log(Y) = 1.26log(X)-0.87. The solid blue line is the phylogenetic regression of aquatic mammals: (A) log(Y) = −0.03log(X)-2.96, (B) log(Y) = 0.11log(X)+-0.50 and (C) log(Y) = −0.07log(X)-0.02. Insert: intercept values and confidence intervals (CI) for aquatic (A) and terrestrial (T) species. Values were calculated from phylogenetic least squares (PGLS) regression analyses applied to 1000 alternative resolutions of the mammalian phylogeny. Error bars represent CI's for the intercept values and are calculated using the standard error (SE) multiplied by 1.96.

We found there was no significant difference (confidence intervals overlap 0) in intercept between terrestrial and aquatic carnivores for minimum (CI -2.22, 1.54; Fig. 1A), maximum (CI -2.48, 1.47; Fig. 1B) and range of prey mass (CI -2.70, 1.00; Fig. 1C). There was a significant difference (confidence intervals do not overlap 0) in slope between terrestrial and aquatic carnivores for minimum (CI 0.37, 1.93), maximum (CI 0.24, 1.94) and range of prey mass (CI 0.49, 2.16). Despite the negative slopes of the aquatic regression lines, these values were not significantly different from 0 for minimum, maximum or prey mass range. The terrestrial regression slopes were positive and significantly different from 0 for minimum, maximum or prey mass range (Fig. 1A, B and C).

### Variance Components Analysis

Order, family and genus explained the maximum proportion of variance in prey mass of carnivores (58–73%; Table 3), providing additional support for the strong influence of phylogenetic relatedness on the prey mass consumed by carnivorous mammals. Body mass of the carnivore explained a relatively large degree of variance (32–39%), where environment had little influence over the mass of prey consumed (<0.01–3%).

**Table 3:** Variance components analysis of prey mass across 108 carnivorous mammal species.

Prey Mass	Variance Source	Variance Component	Total Variance Explained (%)
Minimum	Total	3.17	100
	Order	0.18	5.65
	Family	1.13	35.65
	Genus	0.52	16.65
	Mass	1.23	38.83
	Environment	0.11	3.43
Maximum	Total	3.31	100
	Order	0.43	13.79
	Family	0.19	6.26
	Genus	1.64	52.60
	Mass	1.05	33.70
	Environment	<0.01	<0.01
Range	Total	3.50	100
	Order	0.40	11.33
	Family	0.17	4.88
	Genus	1.81	51.69
	Mass	1.12	31.97
	Environment	<0.01	<0.01

Categories include minimum prey mass (smallest prey size consumed), maximum prey size (largest prey size consumed) and range of prey mass (maximum minus minimum prey mass).

### Descriptive Statistics

Examining the descriptive statistics, the minimum prey mass distribution across all mammals is positively skewed (skew = 0.11), while maximum and range of prey mass is negatively skewed (−0.40 to −0.37; Table S1). The prey mass data has a normal distribution for minimum, maximum and range of prey mass with kurtosis values between 0.62 and 1.18 (Table S1).

**Figure 2:**
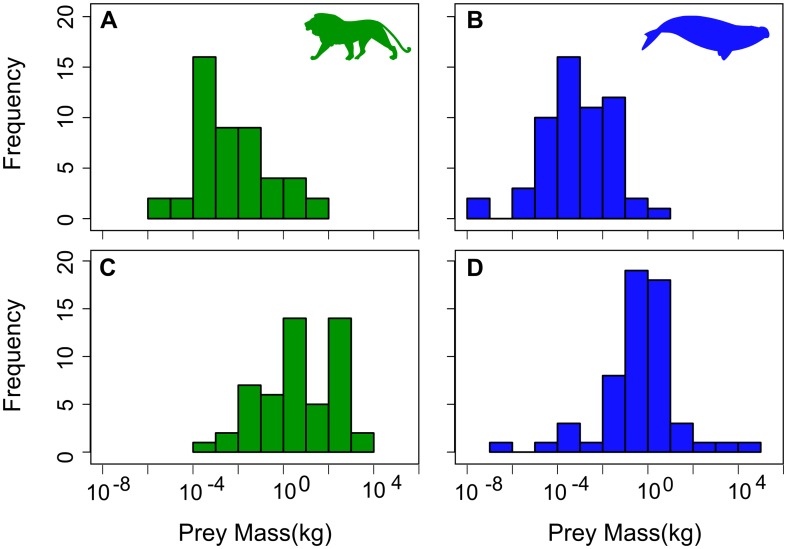
Distributions of the minimum prey mass for (A) terrestrial carnivorous mammals (green bars) and (B) aquatic carnivorous mammals (blue bars), and the maximum prey mass for (C) for terrestrial carnivorous mammals (green bars) and (D) for aquatic carnivorous mammals (blue bars). Silhouettes by uncredited and Chris Huh were downloaded from http://phylopic.org.

When examining the aquatic and terrestrial prey mass distributions, we find that aquatic carnivores tend to feed on smaller-sized prey, with mean prey mass for minimum, maximum and the range being lower than terrestrial carnivores (Fig. 2, Table S2 and S3). Aquatic carnivore prey mass distributions are all negatively skewed (−0.39 to −0.63), but in terrestrial carnivores negatively-skewed distribution are only seen for maximum and range prey mass distributions (−0.25 and −0.35). The prey mass distribution for terrestrial carnivores is relatively flat as suggested by the negative kurtosis values (−0.25 to −0.75). For aquatic species, the prey mass distributions are normally distributed with kurtosis value between 0.88 and 3.17. Additionally, aquatic carnivores feed on prey spanning 12 900 kg, compared with 2 700 kg for terrestrial carnivores.

## Discussion

The best model of prey mass evolution includes both carnivore mass and environment, although environment explains a small percentage (∼8%) of variance in prey size. In spite of this, aquatic and terrestrial mammalian carnivores have different relationships suggesting different optimal foraging strategies. Aquatic mammalian carnivores have no relationship between prey (neither minimum nor maximum) and predator body mass, unlike terrestrial mammalian carnivores where there is a positive prey-predator (both minimum and maximum) body mass relationship. In contrast to terrestrial predators, larger marine carnivores do not have to actively pursue prey with large body mass to meet their energetic requirements [13]. The abundance of small-sized prey in aquatic and marine environments (Fig. 2) is likely to have driven these patterns in marine carnivores. As well as prey availability, it is also important to note the effect of dimensionality on predator-prey relationships and consumption rates. In 3D environments, it has been demonstrated that consumption rates are higher, not only the baseline rates but also the scaling exponent [34]. This not only has an impact on predator-prey relationships (e.g. larger consumer-resource body mass ratios) but also the strength of interactions between trophic levels and the stability of the community.

In addition to the effect of differences in prey availability and dimensionality, there are differences in body size patterns across aquatic and terrestrial carnivores. Marine mammals tend to have larger body sizes compared to terrestrial mammals because of the relaxation of biomechanical constraints and increased thermoregulatory constraints [35]. This has an effect on the predator-prey relationships, which tend to be more accentuated in the marine environment due to this disparity in body size between predators and their prey. This has been illustrated by the presence of larger predator-prey mass ratios in aquatic environments [14], [36].

The lack of relationship between prey mass and predator mass could be because predator morphology is a large driver behind the prey choice of aquatic mammalian carnivores. Morphology, such as gape size, is a limiting factor for aquatic carnivores physically unable to capture larger-sized prey (e.g. altered morphology of appendages to aid with swimming rather than grasping). Additionally, gape limitation in aquatic systems is believed to impact the number of trophic levels that predators can feed from [37]. This is due to aquatic ecosystems being size structured, where body size generally increases with trophic level [38]. Dentition is also likely to be important as carnivores with highly overlapping ranges often have different tooth morphology driven by competition for resources [39]. In the aquatic system mammals have specialised feeding morphology including keratinized baleen plates for filter feeding (mysticete whales), multi-cuspidate interlocking teeth for krill sieving (e.g. crabeater and leopard seals), simple teeth with reduced serration for catching fish (piscivory e.g. dolphins), reduced teeth (e.g. Ross seal), rounded teeth (e.g. walrus) for eating hard-shelled molluscs or even the loss of teeth (e.g. sperm whale and beaked whales) for eating soft-bodied molluscs. Having specialised dentition can minimise resource competition; however it can leave these specialist carnivores vulnerable to higher extinction pressure if their prey populations were to collapse or become extinct [40], [41].

The addition of environment into the PGLS models explains greater variance and has the highest phylogenetic signal only for minimum prey mass and not for models of maximum or range in prey mass. This suggests there are differences between aquatic and terrestrial mammalian carnivores in the patterns of minimum prey body mass only. There are a great number of large aquatic mammalian carnivores feeding on small-sized prey whereas all large terrestrial mammalian carnivores are tied to feeding upon large-sized prey to maximise their energetic intake while minimising their expenditure [13]. In aquatic environments, particularly the marine system, the combination of the high abundance of prey below 500 g, and the schooling nature of these prey, makes it efficient for large carnivores to switch to feeding on smaller prey.

The combined results from the PGLS (phylogenetic signal; λ) and the variance components analysis suggest that phylogenetic relatedness is a major influence of prey mass distribution patterns across carnivorous mammals. The driving factor behind this result are the baleen whales, as they represent closely related species that share a common feeding strategy. This group represents some of the largest species today (up to 200 tonnes) and they feed on some of the smallest prey species (e.g. zooplankton). Baleen whales consist of species from Balaenopteridae and Balaenidae, and all use filter feeding to capture their prey. There are also other feeding strategies, such as pack hunting, that are generally shared across taxonomic levels (i.e. at the family and genus level).

The most likely reason behind the scatter present in the relationship between body mass and prey mass of terrestrial carnivores (Fig. 1A–C), is related to carnivore feeding strategy. Terrestrial carnivores following two feeding strategies: large prey consumers or small prey consumers [13], [42]. Terrestrial carnivore species weighing 21.5 kg or less feed on invertebrates and small vertebrate prey species (<10 kg) [42]. Above the 21.5 kg threshold, carnivores must shift to feeding on large vertebrate prey to meet their energetic requirements [42]. Several carnivore species that feed upon large vertebrate prey have evolved cooperative or pack hunting strategies, which confer several benefits. One benefit of hunting in a pack is the minimisation of the energy expended whilst hunting, but also maximising the size of the prey captured and prey capture efficiency [43], [44]. An increase in the size of the prey captured, energetic intake and hunting success also follows an increase in the number of individuals within the group [43]. Another advantage of hunting cooperatively is that individuals may gain other benefits including increased body size or reproductive success. For example, individual male fosa (*Cryptoprocta ferox*) that forage in groups tend to have larger body size, which also enables increased competition and mating success [45].

There are various drivers influencing the patterns of prey mass consumed by carnivorous mammals. The size of prey chosen by carnivores is driven by the trade-off between energy acquisition and expenditure, the available ecological niches and the dimensionality of the environment [34]. Foraging animals minimise their energetic costs while maximising energetic gains by moving towards an optimal foraging strategy. As different species evolved different foraging strategies, driven by their evolutionary history and environmental influences, this shapes the patterns of prey mass that are consumed by carnivores. Additionally, the mass of prey utilised by carnivores is influenced by the ecological niches available to them and the resource encounter rate, both of which have an effect on the foraging strategy and the prey availability [46]–[48].

Carnivore energetic costs and prey density both influence the minimum prey mass consumed. Carnivores tend to feed on prey above a certain mass due to the increasing inefficiency of feeding on small prey, because a low capture rate will arise when carnivores forage on prey much smaller than themselves [9]. However, this can be overcome in instances where prey species are in high densities, as illustrated by marine carnivores (e.g. baleen whales) who can survive feeding upon prey less than 1 g (e.g. krill and other invertebrates). This is further highlighted by the lower minimum prey mass of aquatic carnivores compared to that of terrestrial carnivores (Fig. 2).

On the other end of the scale, maximum prey mass is driven by morphological constraints (i.e. gape and locomotion) and energetic costs. A carnivore feeding on prey considerably larger than their own mass will result in a mismatch of reaction time, where the carnivore will respond at a slower rate than that of the prey and will end with the prey escaping and the carnivore in an energetic deficit [9]. Additionally, while it would be ideal for all carnivores to feed on large-sized prey species (providing all carnivores could successfully capture large prey), it would be considerably costly from an energetic perspective, not to mention the increased amount of time spent processing and ingesting the prey [13], [49].

Modal prey mass is influenced by environmental drivers. Based on the data used in the study, the minimum prey mass range (0.0001 to 0.01 kg) is the most commonly utilised by carnivorous species from the aquatic and terrestrial environments. The type of prey included within this weight range are invertebrates (aquatic and terrestrial), small mammals, fish and squid. The most common maximum prey range differs between environments, with terrestrial carnivores predominantly feeding on prey between 1 to 100 kg (i.e. small and large mammal prey), compared with 0.01 to 1 kg for aquatic carnivores (i.e. invertebrate, squid and fish prey). With the prey-weight categories of 0.0001–0.01 kg, 1–100 kg and 0.01–1 kg being the most abundant, this suggests that feeding within these ranges is a common foraging strategy across carnivores. Additionally, modal prey mass will also be driven by the characteristics of the environment, where primary productivity and trophic interactions will shape the size of prey available and the abundance of these prey species. For example, productivity within the marine environment is driven by small, single-celled organisms, allowing higher availability of productivity to consumers and higher predator-prey body mass ratios [14].

In summary, carnivorous mammals within our sample differ in the size of prey they consume and this is influenced by a suite of factors including phylogenetic relatedness, carnivore body mass, the characteristics of the environment in which the carnivore resides, and has evolved within, as well as carnivore energetics. Whilst phylogenetic relatedness and carnivore body mass are the dominant drivers of the prey mass consumed by mammals, the physical environment has a role in the minimum-size prey that can be consumed. Previous research has shown that there is a positive relationship between carnivore mass and the mass of their prey. However, we have demonstrated that this is not the case for aquatic mammalian carnivores. Differences in environmental characteristics including primary productivity and food-web structure are driving the differences in prey mass consumed across aquatic and terrestrial carnivores.

Optimal foraging strategies in mammalian carnivores differ not only across species but also physical environments, which needs to be accounted for when thinking about carnivore behaviour. Gaining a better understanding of the relationship between mammalian carnivores and their prey, predator strategies and the factors driving these patterns, aids with predictions of the susceptibility of carnivores to population declines and the role of carnivores within ecosystems.

## Supporting Information

Table S1 Descriptive statistics for the prey mass distributions across 108 carnivorous mammals.
(PDF)

Table S2 Descriptive statistics for the prey mass distributions across 51 carnivorous terrestrial mammals.
(PDF)

Table S3 Descriptive statistics for the prey mass distributions across 57 carnivorous aquatic mammals.
(PDF)

Appendix S1 Database of predator body mass values and prey body mass values, including sources.
(PDF)
