# Supplementary material for: Examining the Prey Mass of Terrestrial and Aquatic Carnivorous Mammals: Minimum, Maximum and Range
Source: PLoS One. 2014 Aug 27;9(8):e106402. doi: 10.1371/journal.pone.0106402 (PMC4146607; doi:10.1371/journal.pone.0106402)
Supplement: Table S3 — Descriptive statistics for the prey mass distributions across 57 carnivorous aquatic mammals. (PDF) [file pone.0106402.s003.pdf]

Table S3 Descriptive statistics for the prey mass distributions across 57 carnivorous aquatic mammals. Minimum is the minimum prey mass consumed, maximum is the maximum prey mass consumed and range is the total range of prey mass consumed (maximum minus minimum).

Prey Mass	Skew	Kurtosis	Median	Mean	Mode	S.D.	Min	Max	Range
Minimum	-0.39	0.88	-3.22	-3.12	-3.30	1.52	-7.52	0.53	8.05
Maximum	-0.59	3.17	-0.11	-0.33	0.64	1.65	-6.10	4.11	10.21
Range	-0.63	3.10	-0.11	-0.36	-1.30	1.68	-6.11	4.11	10.22
