# Supplementary material for: Examining the Prey Mass of Terrestrial and Aquatic Carnivorous Mammals: Minimum, Maximum and Range
Source: PLoS One. 2014 Aug 27;9(8):e106402. doi: 10.1371/journal.pone.0106402 (PMC4146607; doi:10.1371/journal.pone.0106402)
Supplement: Appendix S1 — Database of predator body mass values and prey body mass values, including sources. (PDF) [file pone.0106402.s004.pdf]

Appendix S1 Database of predator body mass values and prey body mass values, including sources.

Tucker & Rogers: Examining the prey mass of terrestrial and aquatic carnivorous mammals: minimum, maximum and range.

Species	log10 Minimum Prey Mass (kg)	log 10 Maximum Prey Mass(kg)	log10 Prey Mass Range (kg)	log10 Predator Mass (kg)	Environment	Prey Mass Souce	Predator Diet Source
Arctocephalus_gazella	-3.222	0.647	0.647	2.532	Marine	Ross et al. 2004; Culik et al. 1994	Ciaputa et al 2006
Arctocephalus_tropicalis	-3.222	-0.493	-0.493	2.520	Marine	Ross et al. 2004; Nel et al. 2001	Ciaputa et al 2006; Robinson et al. 2003
Balaena_mysticetus	-5.000	-0.088	-0.088	5.492	Marine	Pomerleau et al. 2011	Pomerleau et al. 2011
Balaenoptera_acutorostrata	-4.641	-1.301	-1.301	3.747	Marine	Mizdalski 1988; Cardinale & Arrhernius, 2000	Smout et al. 2007; Weslawski et al. 2006
Balaenoptera_borealis	-4.641	-1.301	-1.301	4.345	Marine	Mizdalski 1988; Cardinale & Arrhernius, 2000	Flinn et al. 2002
Balaenoptera_edeni	-4.469	-0.426	-0.426	4.301	Marine	Gomez-Gutierrez et al. 1996; Cervigon et al. 1992; Froese and Pauly, 2011	Froese and Pauly, 2011; Hart et al. 1973; Tershy 1992; Weslawski et al. 2006
Balaenoptera_musculus	-5.117	-3.222	-3.227	5.188	Marine	Omori 1969; Ross et al. 2004	Mizdalski 1988; Fiedler 1998
Balaenoptera_physalus	-4.469	-1.564	-1.564	4.677	Marine	Gomez-Gutierrez et al. 1996; Van Pelt et al. 1997	Tershy et al. 1993; Weslawski et al 2006
Berardius_arnuxii	-1.538	-0.438	-0.474	3.845	Marine	Walker 2002	Jefferson et al. 1993; Culik 2010
Berardius_bairdii	-1.538	-0.438	-0.474	4.056	Marine	Walker 2002	Walker et al. 2002
Callorhinus_ursinus	-3.620	0.216	0.216	1.744	Marine	Kurle et al. 2001	Kurle et al. 2001
Caperea_marginata	-7.042	-3.222	-3.222	4.505	Marine	Mizdalski 1988; Ross et al. 2004	Kemper et al. 2002
Cephalorhynchus_commersonii	-1.602	-1.387	-1.796	1.860	Marine	Clarke et al .1994	Clarke et al .1994
Cephalorhynchus_heavisidii	-0.398	0.559	0.509	1.875	Marine	Augustyn 1990; Eder et al. 2005	Sekiguchi et al. 1992; Elwin et al. 2006; Eder et al. 2005
Cephalorhynchus_hectori	-4.000	0.108	0.108	1.699	Marine	Rayment & Webster 2009	Rayment & Webster 2009
Cystophora_cristata	-4.469	0.845	0.845	3.565	Marine	Gomez-Gutierrez et al. 1996; Julshamn et al. 2004	Hammil 2000
Delphinapterus_leucas	-3.301	-1.476	-1.483	4.176	Marine	Weslawski et al. 2006; Welch et al. 1993	Dahl et al. 2000
Delphinus_delphis	-1.602	0.163	0.156	1.899	Marine	Bykov 1983 ; Eder et al. 2005	Romero et al. 2011
Enhydra_lutris	-2.564	1.079	1.079	2.439	Marine	Kvitek et al. 1992; Harrison et al. 1965	Wolt et al. 2012
Erignathus_barbatus	-4.301	-3.301	-3.347	2.447	Marine	Weslawski et al. 2006	Hjelset et al. 1990
Eschrichtius_robustus	-5.921	-1.367	-1.367	4.238	Marine	Weslawski et al 2006; Pritchard et al. 1979	Dunham et al. 2002
Eubalaena_glacialis	-7.523	-6.097	-6.114	4.602	Marine	Cohen et al. 1981; Weslawski et al. 2006	Mayo et al. 1990
Eumetopias_jubatus	-3.620	0.020	0.019	3.436	Marine	Kurle et al. 2001	Sinclair et al. 2002; Womble et al. 2006
Feresa_attenuata	-2.959	-0.112	-0.113	2.230	Marine	Santos et al. 1997; Santos et al. 1998	Zerbini et al. 1997

Globicephala_melas	-2.347	0.357	0.356	3.097	Marine	Jackson et al. 2000	Clarke et al. 1994
Halichoerus_grypus	-2.921	0.643	0.643	2.920	Marine	Van Pelt et al. 1997; Kjesbu 1989	Hammill et al. 2000; Weslawski et al. 2006; Vilhjálmsson et al. 2002; Van Pelt et al. 1997
Hydrurga_leptonyx	-3.300	2.305	2.305	3.536	Marine	Mizdalski 1988; Adam 2005	Hall Apsland et al. 2004
Hyperoodon_ampullatus	-2.921	-0.072	-0.073	3.826	Marine	Pusineri et al. 2007	Hooker et al. 1999
Kogia_breviceps	-3.398	0.316	0.316	2.560	Marine	West et al. 2009	West et al. 2009
Lagenorhynchus_obliquidens	-1.658	-0.559	-0.595	2.079	Marine	Vilhjalmsson 2002; Kurle et al. 2001	Morton et al. 2000
Leptonychotes_weddellii	-3.300	0.011	0.011	3.615	Marine	Mizdalski 1988; Artigues et al. 2003	Casaux et al. 2006; O'Droscoll et al. 2011; Kock et al 2008; Artigues et al. 2003
Lissodelphis_borealis	-2.854	-1.097	-1.105	2.061	Marine	Watanabe et al. 1999	Jefferson et al. 2003
Lobodon_carcinophaga	-3.300	-0.919	-0.921	3.398	Marine	Mizdalski 1988; Artigues et al. 2003	Artigues et al. 2003; Huckstadt et al. 2012; Mizdalski 1988
Lontra_canadensis	-4.602	0.643	0.643	1.973	Marine	Miron et al. 1990; Kjesbu 1989	Reid et al. 1994; Noguchi et al. 1991; Palace et al. 2001
Megaptera_novaeangliae	-4.653	-4.459	-4.903	5.518	Marine	Mizdalski 1988; Omori 1969	Friedlaender et al. 2008; Mizdalski 1988
Mesoplodon_carlhubbsi	-1.097	-0.479	-0.599	3.176	Marine	Clarke et al. 1998	MacLeod et al. 2003
Mesoplodon_densirostris	-3.854	0.362	0.362	3.000	Marine	Childress 1971; Nakamura and Parin, 1993	Clarke et al. 1994; MacLeod et al. 2003; Belman et al. 1976; Ruiz-Capilla et al. 2001
Mesoplodon_layardii	-1.449	-0.247	-0.275	3.114	Marine	MacLeod et al. 2003	MacLeod et al. 2003
Mesoplodon_mirus	-1.983	-1.018	-1.067	3.146	Marine	MacLeod et al. 2003; Beasly et al. 2013	McLeod et al. 2003; Clarke et al. 1994
Mirounga_angustirostris	-3.300	1.732	1.732	4.084	Marine	Mizdalski 1988; Kohler et al. 1995	Antonelis et al. 1987; Condit et al. 1984
Mirounga_leonina	-3.300	-0.100	-0.100	4.182	Marine	Mizdalski 1988; Slip 1995	Slip 1995
Monodon_monoceros	-3.155	-0.509	-0.510	4.114	Marine	Weslawski et al. 1994; Lucassen et al. 2006	Finley et al 1982
Ommatophoca_rossii	-2.046	-0.060	-0.064	2.301	Marine	Skinner et al. 1994	Skinner et al. 1994
Orcinus_orca	0.527	3.747	3.747	4.752	Marine	Artigues et al. 2003; Jones et al. 2009	Pitman et al. 2003
Otaria_flavescens	-3.699	-0.366	-0.366	3.371	Marine	Clausen et al. 2003	Thompson et al. 1998; Eder et al. 2005; Clarke et al. 1994; Clausen et al. 2003
Phoca_vitulina	-2.824	-0.697	-0.700	2.498	Marine	Bowen et al. 1994	Bowen et al. 1994
Phocarctos_hookeri	-2.699	0.787	0.786	3.107	Marine	Meynier et al. 2009	Meynier et al. 2009
Phocoena_phocoena	-3.301	0.580	0.580	2.740	Marine	Smith 1963; Julshamn et al. 2004	Santos et al. 2004
Physeter_catodon	-1.301	0.248	0.236	4.428	Marine	Gaskin et al. 1967	Gaskin et al. 1967
Pontoporia_blainvillei	-2.796	-0.824	-0.829	1.473	Marine	Santos et al. 1998; Bolasina	Rodriguez et al. 2002

						2006	
Sotalia_fluviatilis	-1.301	-0.767	-0.917	2.740	Marine	Godoy et al. 2002; Lapa-Guimaraes et al. 2005	Borobia et al. 1989
Sotalia_guianensis	-1.523	-0.767	-0.850	2.903	Marine	Godoy et al. 2002; Lapa-Guimaraes et al. 2005	Godoy et al. 2002; Lapa-Guimares et al. 2005; Di Beneditto et al. 2007
Tursiops_truncatus	-3.301	0.643	0.643	3.556	Marine	Smith 1969; Kjesbut 1989	Santos et al. 2001; Skog et al. 2003; Kjesbu et al. 1989; Anderson et al. 2005
Ursus_maritimus	-0.783	4.114	4.114	2.486	Marine	Taylor 1994; Jones et al. 2009	Iverson et al. 2013
Zalophus_californianus	-1.745	1.732	1.732	2.927	Marine	Hunter et al. 1980; Kohler et al. 1995	Lowry et al. 1991; Hunter et al. 1980; Williamson et al. 1984; Quitral et al. 2009
Ziphius_cavirostris	-2.745	0.541	0.541	3.470	Marine	Pusineri et al. 2007; Lordan et al. 2001	Santos et al. 2001; Pusineri et al. 2007; Xavier et al. 2002
Pusa_hispida	-4.000	-2.000	-2.004	1.852	Marine	Weslawski et al. 1994	Weslawski et al. 1994
Acinonyx_jubatus	-0.301	2.740	2.740	1.699	Terrestrial	Hayward et al. 2006	Hayward et al. 2006
Bassariscus_sumichrasti	NA	0.363	NA	-0.125	Terrestrial	Jones et al. 2009	Estrada et al. 1985
Canis_adustus	-5.614	0.415	0.415	0.932	Terrestrial	Redford et al. 1984; Jones et al. 2009	Atkinson et al. 2002
Canis_aureus	-1.714	-0.234	-0.249	0.995	Terrestrial	Jones et al. 2009	Jaeger et al. 2007
Canis_latrans	-0.127	2.665	2.664	1.128	Terrestrial	Jones et al. 2009	Theberge et al. 1989
Canis_lupus	0.582	2.796	2.793	1.633	Terrestrial	Jones et al. 2009	Metz et al. 2012; Nowak et al. 2011
Canis_simensis	-1.135	2.791	2.791	1.161	Terrestrial	Jones et al. 2009	Marino et al . 2010
Cerdocyon_thous	-1.602	-0.006	-0.017	1.114	Terrestrial	Wolcott 1978; Jones et al. 2009	Gatti et al. 2006
Crocuta_crocuta	1.021	3.186	3.183	0.760	Terrestrial	Jones et al. 2009	Hayward et al. 2006
Eira_barbara	-2.699	-0.125	-0.126	1.813	Terrestrial	Paoletti et al. 2000; Jones et al. 2009	Presley et al 2000
Caracal_caracal	-3.457	1.194	1.194	0.650	Terrestrial	Varma et al. 1990; Jones et al. 2009	Avenant et al. 2002
Felis_catus	-3.631	0.544	0.544	0.531	Terrestrial	Brooks et al. 1996. ; Kutt 2012	Kutt 2012; Paltridge et al. 1997
Felis_chaus	-3.545	-0.860	-0.861	0.817	Terrestrial	de los Sanots Gomez 2013; Jones et al. 2009	Mukherjee et al. 2004; Mohammad 2008
Genetta_tigrina	NA	0.687	NA	0.332	Terrestrial	Jones et al. 2009	Ray et al. 2001; Sanchez et al. 2009
Gulo_gulo	-2.147	2.682	2.682	1.333	Terrestrial	Jones et al. 2009	Myhre et al. 1975
Leopardus_pardalis	-2.097	0.602	0.601	1.000	Terrestrial	Meza et al. 2002; Jones et al. 2009	Wang 2002
Leopardus_wiedii	-1.367	0.579	0.574	0.560	Terrestrial	Jones et al. 2009	Wang 2002
Leptailurus_serval	-3.222	-0.652	-0.653	1.079	Terrestrial	Defoliarte 1995; Jones et al. 2009	Thiel 2011
Lycaon_pictus	0.246	2.750	2.749	1.431	Terrestrial	Jones et al. 2009	Hayward et al. 2006

Lynx_canadensis	-1.714	1.258	1.257	1.009	Terrestrial	Jones et al. 2009	Poole 2003
Lynx_lynx	-2.037	2.682	2.682	1.477	Terrestrial	Jones et al. 2009	Odden et al. 2006
Lynx_pardinus	NA	2.382	NA	1.043	Terrestrial	Jones et al. 2009	Gil-Sanchez et al. 2006
Lynx_rufus	-2.180	0.460	0.459	1.055	Terrestrial	Robertson et al. 1982	Delibes et al. 1988
Martes_foina	-2.866	1.676	1.676	0.255	Terrestrial	Speiser et al. 2001; Jones et al. 2009	Bakaloudis et al. 2011
Martes_martes	-3.721	2.167	2.167	0.394	Terrestrial	Clark et al. 1973; Jones et al. 2009	Caryl 2008
Martes_pennanti	-1.714	2.167	2.167	0.502	Terrestrial	Jones et al. 2009	Giuliano et al. 1989
Meles_meles	-2.727	-1.058	-1.067	0.972	Terrestrial	Ma et al. 1993; Jones et al. 2009	Virgos et al. 2004; Goszczynski et al.2000
Mungos_mungo	-3.222	-2.456	-2.538	0.158	Terrestrial	Defoliarte 1995; Dejean et al. 2001	Rood 1975
Mustela_erminea	-3.921	-1.467	-1.469	-0.568	Terrestrial	Martinoli et al. 2001	Martinoli et al. 2001
Mustela_frenata	-1.738	0.407	0.404	-0.823	Terrestrial	Jones et al. 2009	Wilson et al. 1996
Mustela_nivalis	-1.684	-1.500	-1.963	-1.055	Terrestrial	Jones et al. 2009	Jedrzejewska et al. 1995
Mustela_putorius	-2.585	-1.570	-1.614	-0.092	Terrestrial	Jones et al. 2009	Lode 1997
Neovison_vison	-3.509	0.272	0.272	0.037	Terrestrial	Coutts et al. 1973; Wright 1990	Jedrzejewska et al. 2001
Nasua_nasua	-3.222	0.041	0.041	0.672	Terrestrial	Defoliarte 1995; Jones et al. 2009	Alves-Costa et al. 2004
Nyctereutes_procyonoides	-2.727	-1.358	-1.376	0.783	Terrestrial	Ma et al. 1993; Jones et al. 2009	Baltrunaite 2002
Otocyon_megalotis	-3.222	-3.117	-3.785	0.618	Terrestrial	Defoliarte 1995;	Kuntzsch et al. 1992
Panthera_leo	1.653	2.740	2.703	2.312	Terrestrial	Hayward et al. 2005	Hayward et al. 2005
Panthera_onca	0.305	2.464	2.461	1.924	Terrestrial	Jones et al. 2009	Weckel et al. 2006
Panthera_pardus	0.000	2.635	2.634	1.685	Terrestrial	Hayward et al. 2006	Hayward et al. 2006
Panthera_tigris	0.699	3.435	3.434	2.049	Terrestrial	Hayward et al. 2012	Hayward et al. 2012
Procyon_lotor	-3.883	0.653	0.653	1.028	Terrestrial	Smith 2002; Delapane et al. 1999	Schoonover et al. 1951
Proteles_cristata	-3.222	-2.405	-2.476	1.000	Terrestrial	Defoliarte 1995; Punzo 1998	deVries et al. 2011
Puma_concolor	-0.445	1.325	1.318	1.954	Terrestrial	Jones et al. 2009	Moreno et al. 2006
Spilogale_putorius	-3.222	0.082	0.082	-0.222	Terrestrial	Defoliarte 1995; Jones et al. 2009	Munoz-Garcia et al 2005; Crabb 1941
Taxidea_taxus	-5.319	-0.488	-0.488	0.935	Terrestrial	Magnarelli 1983; Jones et al. 2009	Michiner et al. 2004; Sovada et al 1999
Vulpes_cana	-3.222	0.311	0.311	-0.013	Terrestrial	Defoliarte 1995; Jones et al. 2009	Geffen et al. 1992
Vulpes_lagopus	-4.169	2.038	2.038	0.698	Terrestrial	Ring et al. 1980; Jones et al. 2009	Haim et al. 2004; Elmhagen et al. 2000
Vulpes_macrotis	-4.171	0.477	0.477	0.653	Terrestrial	Legaspi et al. 1993; Jones et al. 2009	Munoz-Garcia et al. 2005; List et al. 2003
Vulpes_ruepellii	-3.222	-1.155	-1.159	0.512	Terrestrial	Defoliarte 1995; Jones et al. 2009	Williams et al. 2002

Vulpes_velox	-1.803	1.676	1.676	0.322	Terrestrial	Jones et al. 2009	Sovada et al. 2001
Vulpes_vulpes	-3.635	-1.071	-1.072	0.814	Terrestrial	Warburg 1968; Jones et al. 2009	Jedrzejewski et al. 1992

Appendix S1 References

Adam, P.J. (2005) *Lobodon carcinophaga*. *Mammalian Species*, 1-14.

Alves-Costa, C.P., Da Fonseca, G.A.B. & Christófaro, C. (2004) Variation in the diet of the brown-nosed coati (*Nasua nasua*) in southeastern Brazil. *Journal of Mammalogy*, **85**, 478-482.

Anderson, K.J. & Jetz, W. (2005) The broad-scale ecology of energy expenditure of endotherms. *Ecology Letters*, **8**, 310-318.

Antonelis Jr, G.A., Lowry, M.S., DeMaster, D.P. & Fiscus, C.H. (1987) Assessing northern elephant seal feeding habits by stomach lavage. *Marine Mammal Science*, **3**, 308-322.

Artigues, B., Morales-Nin, B. & Balguerias, E. (2003) Fish length-weight relationships in the Weddell Sea and Bransfield Strait. *Polar Biology*, **26**, 463-467.

Atkinson, R.P.D., Macdonald, D.W. & Kamizola, R. (2002) Dietary opportunism in side-striped jackals *Canis adustus* Sundevall. *Journal of Zoology*, **257**, 129-139.

Augustyn, C. (1990) Biological studies on the chokker squid Loligo vulgaris reynaudii (Cephalopoda; Myopsida) on spawning grounds off the south-east coast of South Africa. *South African Journal of Marine Science*, **9**, 11-26.

Avenant, N.L. & Nel, J.A.J. (2002) Among habitat variation in prey availability and use by caracal *Felis caracal*. *Mammalian Biology - Zeitschrift für Säugetierkunde*, **67**, 18-33.

Bakaloudis, D.E., Vlachos, C.G., Papakosta, M.A., Bontzorlos, V.A. & Chatzinikos, E.N. (2012) Diet Composition and Feeding Strategies of the Stone Marten (*Martes foina*) in a Typical Mediterranean Ecosystem. *The Scientific World Journal*, **2012**

Baltrūnaitė, L. (2002) Diet composition of the red fox (Vulpes vulpes L.), pine marten (Martes martes L.) and raccoon dog (Nyctereutes procyonoides Gray) in clay plain landscape, Lithuania. *Acta Zoologica Lituanica*, **12**, 362-368.

Beasley, I., Cherel, Y., Robinson, S., Betty, E. & Gales, R. (2013) Pygmy sperm whale (Kogia breviceps) stranding record in Tasmania, Australia, and diet of a single specimen. *Papers and Proceedings of the Royal Society of Tasmania* (ed by, p. 25.

Belman, B.W. & Childress, J.J. (1976) Circulatory adaptations to the oxygen minimum layer in the bathypelagic mysid *Gnathophausia ingens*. *Biological Bulletin*, 15-37.

Bolasina, S.N. (2006) Cortisol and hematological response in Brazilian codling, *Urophycis brasiliensis* (Pisces, Phycidae) subjected to anesthetic treatment. *Aquaculture International*, **14**, 569-575.

Borobia, M. & Barros, N.B. (1989) Notes on the diet of marine Sotalia fluviatilis. *Marine Mammal Science*, **5**, 395-399.

Bowen, W. & Harrison, G. (1994) Offshore diet of grey seals Halichoerus grypus near Sable Island, Canada. *Marine ecology progress series. Oldendorf*, **112**, 1-11.

Brooks, S.J., Calver, M.C., Dickman, C.R., Meathrel, C.E. & Bradley, J.S. (1996) Does intraspecific variation in the energy value of a prey species to its predators matter in studies of ecological energetics? A case study using insectivorous vertebrates. *Ecoscience*, **3**, 247-251.

Bykov, V.P. (1983) *Marine Fishes: Chemical composition and processing properties.* Amerind Publishing Co. Pvt. , New Delhi.

Cardinale, M. & Arrhenius, F. (2000) Decreasing weight-at-age of Atlantic herring (Clupea harengus) from the Baltic Sea between 1986 and 1996: a statistical analysis. *ICES Journal of Marine Science: Journal du Conseil*, **57**, 882-893.

Caryl, F.M. (2008) *Pine marten diet and habitat use within a managed coniferous forest*. *PhD Thesis, University of Sterling, Crawley*,

Casaux, R., Baroni, A. & Ramon, A. (2006) The diet of the weddell seal *Leptonychotes weddellii* at the Danco Coast, Antarctic Peninsula. *Polar Biology*, **29**, 257-262.

Cervigón, F., Cipriani, R., Fischer, W., Garibaldi, L., Hendrickx, M., Lemus, A., Márquez, R., Poutiers, J., Robaina, G. & Rodríguez, B. (1992) Fichas FAO de identificación de especies para los fines de la pesca: Guía de campo de las especies comerciales marinas y de aguas salobres de la costa septentrional de Sur América. *Rome, FAO, 513p*,

Childress, J.J. (1971) Respiratory adaptations to the oxygen minimum layer in the bathypelagic mysid Gnathophausia ingens. *The Biological Bulletin*, **141**, 109-121.

Ciaputa, P. & Siciński, J. (2006) Seasonal and annual changes in Antarctic fur seal (*Arctocephalus gazella*) diet in the area of Admiralty Bay, King George Island, South Shetland Islands. *Polish Polar Research*, **27**, 171-184.

Clark, M.G., Bloxham, D.P., Holland, P.C. & Lardy, H.A. (1973) Estimation of the fructose diphosphatase-phosphofructokinase substrate cycle in the flight muscle of< italic> Bombus affinis</italic>. *Biochem. J*, **134**, 589-597.

Clarke, M. & Goodall, N. (1994) Cephalopods in the diets of three odontocete cetacean species stranded at Tierra del Fuego, *Globicephala melaena* (Traill, 1809),

Hyperoodon planifrons (Flower, 1882) and *Cephalorhynchus commersonii* (Lacepede, 1804). *Antarctic Science-Institutional Subscription*, **6**, 149-154.
Clarke, M. & Roeleveld, M. (1998) Cephalopods in the diet of sperm whales caught commercially off Durban, South Africa. *South African Journal of Marine Science*, **20**, 41-45.
Clausen, A. & Pütz, K. (2003) Winter diet and foraging range of gentoo penguins (*Pygoscelis papua*) from Kidney Cove, Falkland Islands. *Polar Biology*, **26**, 32-40.
Cohen, R. & Lough, R. (1981) Length-weight relationships for several copepods dominant in the Georges Bank-Gulf of Maine area. *J. Northw. Atl. Fish. Sci*, **2**, 47-52.
Condit, R. & Le Boeuf, B.J. (1984) Feeding habits and feeding grounds of the northern elephant seal. *Journal of Mammalogy*, 281-290.
Coutts, R.A., Fenton, M.B. & Glen, E. (1973) Food Intake by Captive Myotis lucifugus and Eptesicus fuscus (Chiroptera: Vespertilionidae). *Journal of Mammalogy*, **54**, 985-990.
Crabb, W.D. (1941) Food habits of the prairie spotted skunk in southeastern Iowa. *Journal of Mammalogy*, **22**, 349-364.
Culik, B. (2010) Odontocetes. The toothed whales: "*Berardius bairdii*". UNEP/CMS Secretariat, Bonn, Germany. <http://www.cms.int/reports/small_cetaceans/index.htm> Accessed 5 November 2012.
Culik, B., Wilson, R. & Bannasch, R. (1994) Underwater swimming at low energetic cost by pygoscelid penguins. *Journal of Experimental Biology*, **197**, 65-78.
Dahl, T.M., Lydersen, C., Kovacs, K.M., Falk-Petersen, S., Sargent, J., Gjertz, I. & Gulliksen, B. (2000) Fatty acid composition of the blubber in white whales (*Delphinapterus leucas*). *Polar Biology*, **23**, 401-409.
de los Santos Gómez, A. (2013) Indicating assemblage vulnerability and resilience in the face of climate change by means of adult ground beetle length–weight allometry over elevation strata in Tenerife (Canary Islands). *Ecological Indicators*, **34**, 204-209.
de Vries, J.L., Prik, C.W.W., Bateman, P.W., Cameron, E.Z. & Dalerum, F. (2011) Extension of the diet of an extreme foraging specialist, the aardwolf (*Proteles cristata*). *African Zoology*, **46**, 194-196.
DeFoliart, G.R. (1995) Edible insects as minilivestock. *Biodiversity & Conservation*, **4**, 306-321.
Dejean, A., Suzzoni, J. & Schatz, B. (2001) Behavioral adaptations of an African ponerine ant in the capture of millipedes. *Behaviour*, **138**, 981-996.
Delaplane, K.S. & Hood, W.M. (1999) Economic threshold for Varroa jacobsoni Oud. in the southeastern USA. *Apidologie*, **30**, 383-395.
Delibes, M., Zapata, S.C., Blázquez, M.C. & Rodríguez-Estrella, R. (1997) Seasonal food habits of bobcats (*Lynx rufus*) in subtropical Baja California Sur, Mexico. *Canadian Journal of Zoology*, **75**, 478-483.
Di Beneditto, A.P.M. & Siciliano, S. (2007) Stomach contents of the marine tucuxi dolphin (*Sotalia guianensis*) from Rio de Janeiro, south-eastern Brazil. *Journal of the Marine Biological Association of the United Kingdom*, **87**, 253-254.
Dunham, J.S. & Duffus, D.A. (2002) Foraging patterns of gray whales in central Clayoquot Sound, British Columbia, Canada. *Marine Ecology Progress Series*, **223**, 299-310.
Eder, E. & Lewis, M. (2005) Proximate composition and energetic value of demersal and pelagic prey species from the SW Atlantic Ocean. *Marine Ecology Progress Series*, **291**, 43-52.
Elmhagen, B., Tannerfeldt, M., Verucci, P. & Angerbjörn, A. (2000) The arctic fox (*Alopex lagopus*): an opportunistic specialist. *Journal of Zoology*, **251**, 139-149.
Elwin, S., Meyer, M.A., Best, P.B., Kotze, P.G.H., Thronton, M. & Swanson, S. (2006) Range and movements of female Heaviside's dolphins (*Cephalorhynchus heavisidii*), as determined by satellite-linked telemetry. *Journal of Mammalogy*, **87**, 866-877.
Estrada, A. & Coates-Estrada, R. (1985) A preliminary study of resource overlap between howling monkeys (*Alouatta palliata*) and other arboreal mammals in the tropical rain forest of Los Tuxtlas, Mexico. *American Journal of Primatology*, **9**, 27-37.
Fiedler, P.C., Reilly, S.B., Hewitt, R.P., Demer, D., Philbrick, V.A., Smith, S., Armstrong, W., Croll, D.A., Tershy, B.R. & Mate, B.R. (1998) Blue whale habitat and prey in the California Channel Islands. *Deep Sea Research Part II: Topical Studies in Oceanography*, **45**, 1781-1801.
Finley, K.J. & Gibb, E.J. (1982) Summer diet of the narwhal (*Monodon monoceros*) in Pond Inlet, northern Baffin Island. *Canadian Journal of Zoology*, **60**, 3353-3363.
Flinn, R.D., Trites, A.W., Gregr, E.J. & Perry, R.I. (2002) Diets of fin, sei, and sperm whales in British Columbia: an analysis of commercial whaling records, 1963–1967. *Marine Mammal Science*, **18**, 663-679.
Friedlaender, A.S., Fraser, W.R., Patterson, D., Qian, S.S. & Halpin, P.N. (2008) The effects of prey demography on humpback whale (*Megaptera novaeangliae*) abundance around Anvers Island, Antarctica. *Polar Biology*, **31**, 1217-1224.
Froese, R. & Pauly, D. (2011) FishBase. World Wide Web electronic publication. www.fishbase.org Accessed 15 January 2014.
Gaskin, D.E. & Cawthorn, M.W. (1967) Diet and feeding habits of the sperm whale (*Physeter catodon* L.) in the Cook Strait region of New Zealand. *New Zealand Journal of Marine and Freshwater Research*, **1**, 156-179.
Gatti, A., Bianchi, R., Rosa, C.R.X. & Mendes, S.L. (2006) Diet of two sympatric carnivores, *Cerdocyon thous* and *Procyon cancrivorus*, in a restinga area of Espirito Santo State, Brazil. *Journal of Tropical Ecology*, **22**, 227-230.

Geffen, E., Hefner, R., MacDonald, D.W. & Ucko, M. (1992) Diet and foraging behavior of Blanford's foxes, *Vulpes cana*, in Israel. *Journal of Mammalogy*, 395-402.
Gil-Sánchez, J., Ballesteros-Duperón, E. & Bueno-Segura, J. (2006) Feeding ecology of the Iberian lynxLynx pardinus in eastern Sierra Morena (Southern Spain). *Acta Theriologica*, **51**, 85-90.
Giuliano, W.M., Litvaitis, J.A. & Stevens, C.L. (1989) Prey Selection in Relation to Sexual Dimorphism of Fishers (*Martes pennanti*) in New Hampshire. *Journal of Mammalogy*, **70**, 639-641.
Godoy, E.A.S., Almeida, T.C.M. & Zalmon, I.R. (2002) Fish assemblages and environmental variables on an artificial reef north of Rio de Janeiro, Brazil. *ICES Journal of Marine Science: Journal du Conseil*, **59**, S138-S143.
Gómez-Gutiérrez, J., De Silva-Dávila, R. & Lavaniegos-Espejo, B. (1996) Growth production of the euphausiid *Nyctiphanes simple*x on the coastal shelf off Bahía Magdalena, Baja California Sur, México. *Marine ecology progress series. Oldendorf*, **138**, 309-314.
Goszczyński, J., Jedrzejewska, B. & Jedrzejewski, W. (2000) Diet composition of badgers (*Meles meles*) in a pristine forest and rural habitats of Poland compared to other European populations. *Journal of Zoology*, **250**, 495-505.
Haim, A., Saarela, S., Hohtola, E. & Zisapel, N. (2004) Daily rhythms of oxygen consumption, body temperature, activity and melatonin in the Norwegian lemming *Lemmus lemmus* under northern summer photoperiod. *Journal of Thermal Biology*, **29**, 629-633.
Hall-Aspland, S. & Rogers, T.L. (2004) Summer diet of leopard seals (*Hydrurga leptonyx*) in Prydz Bay, Eastern Antarctica. *Polar Biology*, **27**, 729-734.
Hammill, M. & Stenson, G. (2000) Estimated prey consumption by harp seals (*Phoca groenlandica*), hooded seals (*Cystophora cristata*), grey seals (Halichoerus grypus) and harbour seals (*Phoca vitulina*) in Atlantic Canada. *Journal of Northwest Atlantic Fishery Science*, **26**, 1-24.
Harrison, F. & Martin, A. (1965) Excretion in the cephalopod, Octopus dofleini. *Journal of Experimental Biology*, **42**, 71-98.
Hart, J.L. (1973) Pacific fishes of Canada. *Fisheries Research Board of canada(Bull. 180), Ottawa, Canada. 749*, 1973.
Hayward, M.W. (2006) Prey preferences of the spotted hyaena (*Crocuta crocuta*) and degree of dietary overlap with the lion (*Panthera leo*). *Journal of Zoology*, **270**, 606-614.
Hayward, M.W. & Kerley, G.I.H. (2005) Prey preferences of the lion (*Panthera leo*). *Journal of Zoology*, **267**, 309-322.
Hayward, M., Jędrzejewski, W. & Jędrzewska, B. (2012) Prey preferences of the tiger *Panthera tigris*. *Journal of Zoology*,
Hayward, M., Hofmeyr, M., O'brien, J. & Kerley, G. (2006) Prey preferences of the cheetah (*Acinonyx jubatus*)(Felidae: Carnivora): morphological limitations or the need to capture rapidly consumable prey before kleptoparasites arrive? *Journal of Zoology*, **270**, 615-627.
Hayward, M.W., O'Brien, J., Hofmeyr, M. & Kerley, G.I. (2006) Prey preferences of the African wild dog Lycaon pictus (Canidae: Carnivora): ecological requirements for conservation. *Journal of Mammalogy*, **87**, 1122-1131.
Hayward, M., Henschel, P., O'brien, J., Hofmeyr, M., Balme, G. & Kerley, G. (2006) Prey preferences of the leopard (*Panthera pardus*). *Journal of Zoology*, **270**, 298-313.
Hjelset, A., Andersen, M., Gjertz, I., Lydersen, C. & Gulliksen, B. (1999) Feeding habits of bearded seals (*Erignathus barbatus*) from the Svalbard area, Norway. *Polar Biology*, **21**, 186-193.
Hooker, S.K. & Baird, R.W. (1999) Deep–diving behaviour of the northern bottlenose whale, Hyperoodon ampullatus (Cetacea: Ziphiidae). *Proceedings of the Royal Society of London. Series B: Biological Sciences*, **266**, 671-676.
Hückstädt, L., Burns, J., Koch, P., McDonald, B., Crocker, D. & Costa, D. (2012) Diet of a specialist in a changing environment: the crabeater seal along the western Antarctic Peninsula. *Marine Ecology Progress Series*, **455**, 287.
Hunter, J. & Macewicz, B.J. (1980) Sexual maturity, batch fecundity, spawning frequency, and temporal pattern of spawning for the northern anchovy, *Engraulis mordax*, during the 1979 spawning season. *CalCOFI Rep*, **21**, 139-149.
Iversen, M., Aars, J., Haug, T., Alsos, I.G., Lydersen, C., Bachmann, L. & Kovacs, K.M. (2013) The diet of polar bears (Ursus maritimus) from Svalbard, Norway, inferred from scat analysis. *Polar biology*, **36**, 561-571.
Jackson, G.D., Buxton, N.G. & George, M.J. (2000) Diet of the southern opah Lampris immaculatus on the Patagonian Shelf; the significance of the squid Moroteuthis ingens and anthropogenic plastic. *Marine Ecology Progress Series*, **206**, 261-271.
Jaeger, M.M., Haque, E., Sultana, P. & Bruggers, R.L. (2007) Daytime cover, diet and space-use of golden jackals (*Canis aureus*) in agro-ecosystems of Bangladesh. *Mammalia*, **71**, 1-10.
Jędrzejewska, B., Sidorovich, V.E., Pikulik, M.M. & Jędrzejewska, W. (2001) Feeding habits of the otter and the American mink in Białowieża Primeval Forest (Poland) compared to other Eurasian populations. *Ecography*, **24**, 165-180.
Jędrzejewski, W. & Jędrzejewska, B. (1992) Foraging and diet of the red fox *Vulpes vulpes* in relation to variable food resources in Biatowieza National Park, Poland. *Ecography*, **15**, 212-220.
Jędrzejewski, W., Jędrzejewska, B. & Szymura, L. (1995) Weasel Population Response, Home Range, and Predation on Rodents in a Deciduous Forest in Poland. *Ecology*,

76, 179-195.
Jefferson, T.A., Leatherwood, S. & Webber, M.A. (2003) *FAO species identification guide. Marine mammals of the world.*, Rome.
Jones, K.E., Bielby, J., Cardillo, M., Fritz, S.A., O'Dell, J., Orme, C.D.L., Safi, K., Sechrest, W., Boakes, E.H., Carbone, C., Connolly, C., Cutts, M.J., Foster, J.K., Grenyer, R., Habib, M., Plaster, C.A., Price, S.A., Rigby, E.A., Rist, J., Teacher, A., Bininda-Emonds, O.R.P., Gittleman, J.L., Mace, G.M., Purvis, A. & Michener, W.K. (2009) PanTHERIA: a species-level database of life history, ecology, and geography of extant and recently extinct mammals. *Ecology*, **90**, 2648-2648.
Julshamn, K., Lundebye, A.K., Heggstad, K., Berntssen, M. & Boe, B. (2004) Norwegian monitoring programme on the inorganic and organic contaminants in fish caught in the Barents Sea, Norwegian Sea and North Sea, 1994–2001. *Food additives and contaminants*, **21**, 365-376.
Kemper, C.M. (2002) Distribution of the pygmy right whale, *Caperea marginata*, in the Australasian region. *Marine Mammal Science*, **18**, 99-111.
Kjesbu, O. (1989) The spawning activity of cod, *Gadus morhua* L. *Journal of fish biology*, **34**, 195-206.
Kock, K.H., Pshenichnov, L., Jones, C.D., Gröger, J. & Riehl, R. (2008) The biology of the spiny icefish *Chaenodraco wilsoni* Regan, 1914. *Polar Biology*, **31**, 381-393.
Kohler, N.E., Casey, J.G. & Turner, P.A. (1995) Length-weight relationships for 13 species of sharks from the western North Atlantic. *Fishery Bulletin*, **93**, 412-418.
Kuntzsch, V. & Nel, J. (1992) Diet of bat-eared foxes *Otocyon megalotis* in the Karoo. *Koedoe-African Protected Area Conservation and Science*, **35**, 37-48.
Kurle, C.M. & Worthy, G.A.J. (2001) Stable isotope assessment of temporal and geographic differences in feeding ecology of northern fur seals (*Callorhinus ursinus*) and their prey. *Oecologia*, **126**, 254-265.
Kutt, A.S. (2012) Feral cat (*Felis catus*) prey size and selectivity in north-eastern Australia: implications for mammal conservation. *Journal of Zoology*, **287**, 292-300.
Kvitek, R., Oliver, J., DeGange, A. & Anderson, B. (1992) Changes in Alaskan soft-bottom prey communities along a gradient in sea otter predation. *Ecology*, 413-428.
Lapa-Guimarães, J., de Felício, P.E. & Guzmán, E.S.C. (2005) Chemical and microbial analyses of squid muscle (*Loligo plei*) during storage in ice. *Food chemistry*, **91**, 477-483.
Legaspi, J.C. & O'Neil, R.J. (1993) Life history of Podisus maculiventris given low numbers of Epilachna varivestis as prey. *Environmental entomology*, **22**, 1192-1200.
List, R. & Macdonald, D.W. (2003) Home range and habitat use of the kit fox (*Vulpes macrotis*) in a prairie dog (Cynomys ludovicianus) complex. *Journal of Zoology*, **259**, 1-5.
Lodé, T. (1997) Trophic status and feeding habits of the European Polecat *Mustela putorius* L. 1758. *Mammal Review*, **27**, 177-184.
Lordan, C., Collins, M.A., Key, L.N. & Browne, E.D. (2001) The biology of the ommastrephid squid, Todarodes sagittatus, in the north-east Atlantic. *Journal of the Marine Biological Association of the UK*, **81**, 299-306.
Lowry, M.S., Francis, M., Yochem, P.K., Stewart, B.S. & Heath, C.B. (1991) Seasonal and annual variability in the diet of California sea lions, *Zalophus californianus*, at San Nicolas Island, California, 1981− 86. *Fish. Bull*, **89**, 331-336.
Lucassen, M., Koschnick, N., Eckerle, L.G. & Pörtner, H.-O. (2006) Mitochondrial mechanisms of cold adaptation in cod (*Gadus morhua* L.) populations from different climatic zones. *Journal of Experimental Biology*, **209**, 2462-2471.
Ma, W.-c. & Bodt, J. (1993) Differences in toxicity of the insecticide chlorpyrifos to six species of earthworms (Oligochaeta, Lumbricidae) in standardized soil tests. *Bulletin of Environmental Contamination and Toxicology*, **50**, 864-870.
MacLeod, C., Santos, M. & Pierce, G. (2003) Review of data on diets of beaked whales: evidence of niche separation and geographic segregation. *Journal of the Marine Biological Association of the UK*, **83**, 651-665.
Magnarelli, L.A. (1983) Nectar sugars and caloric reserves in natural populations of *Aedes canadensis* and *Aedes stimulans* (Diptera: Culicidae). *Environmental entomology*, **12**, 1482-1486.
Marino, J., Mitchell, R. & Johnson, P.J. (2010) Dietary specialization and climatic-linked variations in extant populations of Ethiopian wolves. *African Journal of Ecology*, **48**, 517-525.
Martinoli, A., Preatoni, D.G., Chiarenzi, B., Wauters, L.A. & Tosi, G. (2001) Diet of stoats (*Mustela erminea*) in an Alpine habitat:The importance of fruit consumption in summer. *Acta Oecologica*, **22**, 45-53.
Mayo, C.A. & Marx, M.K. (1990) Surface foraging behaviour of the North Atlantic right whale, *Eubalaena glacialis*, and associated zooplankton characteristics. *Canadian Journal of Zoology*, **68**, 2214-2220.
Metz, M.C., Smith, D.W., Vucetich, J.A., Stahler, D.R. & Peterson, R.O. (2012) Seasonal patterns of predation for gray wolves in the multi-prey system of Yellowstone National Park. *Journal of Animal Ecology*, **81**, 553-563.
Meynier, L., Mackenzie, D.D.S., Duignan, P.J., Chilvers, B.L. & Morel, P.C.H. (2009) Variability in the diet of New Zealand sea lion (*Phocarctos hookeri*) at the Auckland Islands, New Zealand. *Marine Mammal Science*, **25**, 302-326.
Meza, A.d.V., Meyer, E.M. & González, C.A.L. (2002) Ocelot (Leopardus Pardalis) Food Habits in a Tropical Deciduous Forest of Jalisco, Mexico. *American Midland Naturalist*, **148**, 146-154.

Michener, G.R. (2004) Hunting techniques and tool use by North American badgers preying on Richardson's ground squirrels. *Journal of Mammalogy*, **85**, 1019-1027.
Miron, G. & Desrosiers, G. (1990) Distributions and population structures of two intertidal estuarine polychaetes in the lower St. Lawrence Estuary, with special reference to environmental factors. *Marine Biology*, **105**, 297-306.
Mizdalski, E. (1988) Weight and length data of zooplankton in the Weddell Sea in austral spring 1986 (ANT V/3). *Berichte zur Polarforschung (Reports on Polar Research)*, **55**
Mohammad, M.K. (2008) The Parasitic Fauna And The Food Habits Of The Wild Jungle Cat *Felis chaus* Furax De Winton, 1898 In Iraq. *Bull. Iraq nat. Hist. Mus*, **10**, 65-78.
Moreno, R.S., Kays, R.W. & Samudio Jr, R. (2006) Competitive release in diets of ocelot (Leopardus pardalis) and puma (Puma concolor) after jaguar (Panthera onca) decline. *Journal of Mammalogy*, **87**, 808-816.
Morton, A. (2000) Occurrence, Photo-Identification and Prey of Pacific White-Sided Dolphins (*Lagenorhyncus obliquidens*) in the Broughton Archipelago, Canada 1984–1998. *Marine Mammal Science*, **16**, 80-93.
Mukherjee, S., Goyal, S.P., Johnsingh, A.J.T. & Leite Pitman, M.R.P. (2004) The importance of rodents in the diet of jungle cat (*Felis chaus*), caracal (*Caracal caracal*) and golden jackal (*Canis aureus*) in Sariska Tiger Reserve, Rajasthan, India. *Journal of Zoology*, **262**, 405-411.
Muñoz, G.A. & Williams, J.B. (2005) Basal metabolic rate in carnivores is associated with diet after controlling for phylogeny. *Physiological and Biochemical Zoology*, **78**, 1039-1056.
Myhre, R. & Myrberget, S. (1975) Diet of Wolverines (*Gulo gulo*) in Norway. *Journal of Mammalogy*, **56**, 752-757.
Nakamura, I. & Parin, N.V. (1993) An Annotated and Illustrated Catalogue of the Snake Mackerels, Snoeks, Escolars, Gemfishes, Sackfishes, Domine, Oilfish, Cutlassfishes, Scabbardfishes, Hairtails, and Frostfishes Known to Date. *FAO Fisheries Synopsis No. 125, Vol. 15*,
Nel, D., Lutjeharms, J., Pakhomov, E., Ansorge, I., Ryan, P. & Klages, N. (2001) Exploitation of mesoscale oceanographic features by grey-headed albatross *Thalassarche chrysostoma* in the southern Indian Ocean. *Marine Ecology Progress Series*, **217**, 15-26.
Noguchi, G.E. & Hesselberg, R.J. (1991) Parental transfer of organic contaminants to young-of-the-year spottail shiners, Notropis hudsonius. *Bulletin of environmental contamination and toxicology*, **46**, 745-750.
Nowak, S., Mysłajek, R.W., Kłosińska, A. & Gabryś, G. (2011) Diet and prey selection of wolves (*Canis lupus*) recolonising Western and Central Poland. *Mammalian Biology - Zeitschrift für Säugetierkunde*, **76**, 709-715.
Odden, J., Linnell, J.C. & Andersen, R. (2006) Diet of Eurasian lynx, *Lynx lynx*, in the boreal forest of southeastern Norway: the relative importance of livestock and hares at low roe deer density. *European Journal of Wildlife Research*, **52**, 237-244.
O'Driscoll, R.L., Macaulay, G.J., Gauthier, S., Pinkerton, M. & Hanchet, S. (2011) Distribution, abundance and acoustic properties of Antarctic silverfish (*Pleuragramma antarcticum*) in the Ross Sea. *Deep Sea Research Part II: Topical Studies in Oceanography*, **58**, 181-195.
Omori, M. (1969) Weight and chemical composition of some important oceanic zooplankton in the North Pacific Ocean. *Marine Biology*, **3**, 4-10.
Palace, V.P., Allen-Gil, S.M., Brown, S.B., Evans, R.E., Metner, D.A., Landers, D.H., Curtis, L.R., Klaverkamp, J.F., Baron, C.L. & Lyle Lockhart, W. (2001) Vitamin and thyroid status in arctic grayling (*Thymallus arcticus*) exposed to doses of 3, 3′, 4, 4′-tetrachlorobiphenyl that induce the phase I enzyme system. *Chemosphere*, **45**, 185-193.
Paltridge, R., Gibson, D. & Edwards, G. (1997) Diet of the Feral Cat (*Felis catus*) in Central Australia. *Wildlife Research*, **24**, 67-76.
Paoletti, M.G., Dufour, D.L., Cerda, H., Torres, F., Pizzoferrato, L. & Pimentel, D. (2000) The Importance of Leaf- and Litter-Feeding Invertebrates as Sources of Animal Protein for the Amazonian Amerindians. *Proceedings: Biological Sciences*, **267**, 2247-2252.
Pitman, R.L. & Ensor, P. (2003) Three forms of killer whales (*Orcinus orca*) in Antarctic waters. *Journal of Cetacean Research and Management*, **5**, 131-140.
Pomerleau, C., Ferguson, S.H. & Walkusz, W. (2011) Stomach contents of bowhead whales (*Balaena mysticetus*) from four locations in the Canadian Arctic. *Polar Biology*, **34**, 615-620.
Poole, K.G. (2003) A review of the Canada lynx, *Lync canadensis*, in Canada. *The Canadian Field- Naturalist*, **117**, 360-376.
Presley, S.J. (2000) Eira barbara. *Mammalian species*, 1-6.
Pritchard, A. & Eddy, S. (1979) Lactate formation in *Callianassa californiensis* and *Upogebia pugettensis* (Crustacea: Thalassinidea). *Marine Biology*, **50**, 249-253.
Punzo, F. (1998) The effects of reproductive status on sprint speed in the solifuge, Eremobates marathoni (Solifugae, Eremobatidae). *Journal of Arachnology*, 113-116.
Pusineri, C., Magnin, V., Meynier, L., Spitz, J., Hassani, S. & Ridoux, V. (2007) Food and feeding ecology of the common dolphin (Delphinus delphis) in the oceanic Northeast Atlantic and comparison with its diet in neritic areas. *Marine Mammal Science*, **23**, 30-47.
Quitral, V., Donoso, M.L., Ortiz, J., Herrera, M.V., Araya, H. & Aubourg, S.P. (2009) Chemical changes during the chilled storage of Chilean jack mackerel (*Trachurus murphyi*): Effect of a plant-extract icing system. *LWT-Food Science and Technology*, **42**, 1450-1454.
Ray, J. & Sunquist, M. (2001) Trophic relations in a community of African rainforest carnivores. *Oecologia*, **127**, 395-408.

Rayment, W. & Webster, T. (2009) Observations of Hector's dolphins (Cephalorhynchus hectori) associating with inshore fishing trawlers at Banks Peninsula, New Zealand.
Redford, K.H. & Dorea, J.G. (1984) The nutritional value of invertebrates with emphasis on ants and termites as food for mammals. *Journal of Zoology*, **203**, 385-395.
Reid, D., Code, T., Reid, A. & Herrero, S. (1994) Food habits of the river otter in a boreal ecosystem. *Canadian Journal of Zoology*, **72**, 1306-1313.
Ring, R.A. & Tesar, D. (1980) Cold-hardiness of the arctic beetle, *Pytho americanus* Kirby Coleoptera, Pythidae (Salpingidae). *Journal of Insect Physiology*, **26**, 763-774.
Robertson, H., Nicolson, S. & Louw, G. (1982) Osmoregulation and temperature effects on water loss and oxygen consumption in two species of African scorpion. *Comparative Biochemistry and Physiology Part A: Physiology*, **71**, 605-609.
Robinson, S., Goldsworthy, S., Van den Hoff, J. & Hindell, M. (2003) The foraging ecology of two sympatric fur seal species, *Arctocephalus gazella* and *Arctocephalus tropicalis*, at Macquarie Island during the austral summer. *Marine and Freshwater Research*, **53**, 1071-1082.
Rodríguez, D., Rivero, L. & Bastida, R. (2002) Feeding ecology of the franciscana (*Pontoporia blainvillei*) in marine and estuarine waters of Argentina. *Latin American Journal of Aquatic Mammals*, **1**, 77-94.
Romero, M.A., Dans, S.L., García, N., Svendsen, G.M., González, R. & Crespo, E.A. (2011) Feeding habits of two sympatric dolphin species off North Patagonia, Argentina. *Marine Mammal Science*, **28**, 364-377.
Rood, J.P. (1975) Population dynamics and food habits of the banded mongoose. *African Journal of Ecology*, **13**, 89-111.
Ross, R.M., Quetin, L.B., Newberger, T. & Oakes, S.A. (2004) Growth and behavior of larval krill (*Euphausia superba*) under the ice in late winter 2001 west of the Antarctic Peninsula. *Deep Sea Research Part II: Topical Studies in Oceanography*, **51**, 2169-2184.
Ruiz-Capillas, C. & Moral, A. (2001) Residual effect of CO2 on hake (*Merluccius merluccius L.*) stored in modified and controlled atmospheres. *European Food Research and Technology*, **212**, 413-420.
Sánchez, M., Rodrigues, P., Ortuño, V. & Herrero, J. (2009) Feeding habits of the genet Genetta genetta in an Iberian continental wetland. *Hystrix, the Italian Journal of Mammalogy*, **19**
Santos, R. & Haimovici, M. (1997) Reproductive biology of winter-spring spawners of *Illex argentinus* (Cephalopoda: Ommastrephidae) off southern Brazil. *Scientia Marina(Barcelona)*, **61**, 53-64.
Santos, R. & Haimovici, M. (1998) Trophic relationships of the long-finned squid *Loligo sanpaulensis* on the southern Brazilian shelf. *South African Journal of Marine Science*, **20**, 81-91.
Santos, M., Pierce, G., Herman, J., Lopez, A., Guerra, A., Mente, E. & Clarke, M. (2001) Feeding ecology of Cuvier's beaked whale (*Ziphius cavirostris*): a review with new information on the diet of this species. *JMBA-Journal of the Marine Biological Association of the United Kingdom*, **81**, 687-694.
Santos, M., Pierce, G., Learmonth, J., Reid, R., Ross, H., Patterson, I., Reid, D. & Beare, D. (2004) Variability in the diet of harbor porpoises (*Phocoena phocoena*) in Scottish waters 1992–2003. *Marine Mammal Science*, **20**, 1-27.
Schoonover, L.J. & Marshall, W.H. (1951) Food Habits of the Raccoon (Procyon lotor hirtus) in North-Central Minnesota. *Journal of Mammalogy*, **32**, 422-428.
Sekiguchi, K., Klages, N. & Best, P. (1992) Comparative analysis of the diets of smaller odontocete cetaceans along the coast of southern Africa. *South African Journal of Marine Science*, **12**, 843-861.
Sinclair, E.H. & Zeppelin, T.K. (2002) Seasonal and spatial differences in diet in the western stock of Steller sea lions (*Eumetopias jubatus*). *Journal of Mammalogy*, **83**, 973-990.
Skinner, J. & Klages, N. (1994) On some aspects of the biology of the Ross seal *Ommatophoca rossii* from King Haakon VII Sea, Antarctica. *Polar Biology*, **14**, 467-472.
Skog, T.E., Hylland, K., Torstensen, B.E. & Berntssen, M.H.G. (2003) Salmon farming affects the fatty acid composition and taste of wild saithe *Pollachius virens* L. *Aquaculture Research*, **34**, 999-1007.
Slip, D.J. (1995) The diet of southern elephant seals (*Mirounga leonina*) from Heard Island. *Canadian Journal of Zoology*, **73**, 1519-1528.
Smith, R.I. (1963) A comparison of salt loss rate in three species of brackish-water nereid polychaetes. *Biological Bulletin*, **125**, 332-343.
Smith, P.W. (2002) *The Fishes of Illinois*. University of Illinois Press, Illinois, pg 152.
Smout, S. & Lindstrøm, U. (2007) Multispecies functional response of the minke whale *Balaenoptera acutorostrata* based on small-scale foraging studies. *Marine Ecology Progress Series*, **341**, 277-291.
Sovada, M.A., Roaldson, J.M. & Sargeant, A.B. (1999) Foods of American badgers in west-central Minnesota and southeastern North Dakota during the duck nesting season. *The American midland naturalist*, **142**, 410-414.
Speiser, B., Zaller, J.G. & Neudecker, A. (2001) Size-specific susceptibility of the pest slugs Deroceras reticulatum and Arion lusitanicus to the nematode biocontrol agent Phasmarhabditis hermaphrodita. *BioControl*, **46**, 311-320.
Taylor, J.R. (1994) Changes in body mass and body reserves of breeding Little Auks (*Alle alle* L.). *Polish Polar Res*, **123**, 149-168.

Tershy, B.R. (1992) Body size, diet, habitat use, and social behavior of Balaenoptera whales in the Gulf of California. *Journal of Mammalogy*, 477-486.
Tershy, B.R., Acevedo, G., Breese, D. & Strong, C.S. (1993) Diet and feeding behavior of fin and Bryde's whales in the central Gulf of California, Mexico. *Rev Inv Cient*, **1**, 31-38.
Theberge, J.B. & Wedeles, C.H.R. (1989) Prey selection and habitat partitioning in sympatric coyote and red fox populations, southwest Yukon. *Canadian Journal of Zoology*, **67**, 1285-1290.
Thiel , C. (2011) *Ecology and population status of the Serval Leptailurus serval (Schriber, 1776) in Zambia*. *PhD Thesis, Universitäts-und Landesbibliothek, Bonn*,
Thompson, D., Duck, C.D., McConnell, B.J. & Garrett, J. (1998) Foraging behaviour and diet of lactating female southern sea lions (*Otaria flavescens*) in the Falkland Islands. *Journal of Zoology*, **246**, 135-146.
Van Pelt, T.I., Piatt, J.F., Lance, B.K. & Roby, D.D. (1997) Proximate composition and energy density of some North Pacific forage fishes. *Comparative Biochemistry and Physiology Part A: Physiology*, **118**, 1393-1398.
Varma, M., Heller-Haupt, A., Trinder, P. & Langi, A. (1990) Immunization of guinea-pigs against Rhipicephalus appendiculatus adult ticks using homogenates from unfed immature ticks. *Immunology*, **71**, 133.
Vilhjálmsson, H. (2002) Capelin (*Mallotus villosus*) in the Iceland–East Greenland–Jan Mayen ecosystem. *ICES Journal of Marine Science: Journal du Conseil*, **59**, 870-883.
Virgós, E., Mangas, J.G., Blanco-Aguiar, J.A., Garrote, G., Almagro, N. & Viso, R.P. (2004) Food habits of European badgers (*Meles meles*) along an altitudinal gradient of Mediterranean environments: a field test of the earthworm specialization hypothesis. *Canadian Journal of Zoology*, **82**, 41-51.
Walker, W.A., Mead, J.G. & Brownell, R.L. (2002) Diets of Baird's beaked whales, *Berardius bairdii*, in the southern sea of Okhotsk and off the pacific coast of Honshu, Japan. *Marine Mammal Science*, **18**, 902-919.
Wang, E. (2002) Diets of Ocelots (*Leopardus pardalis*), Margays (*L. wiedii*), and Oncillas (*L. tigrinus*) in the Atlantic Rainforest in Southeast Brazil. *Studies on Neotropical Fauna and Environment*, **37**, 207-212.
Warburg, M. (1968) Simultaneous measurement of body temperature and water loss in isopods. *Crustaceana*, **14**, 39-44.
Watanabe, H., Moku, M., Kawaguchi, K., Ishimaru, K. & Ohno, A. (1999) Diel vertical migration of myctophid fishes (Family Myctophidae) in the transitional waters of the western North Pacific. *Fisheries Oceanography*, **8**, 115-127.
Weckel, M., Giuliano, W. & Silver, S. (2006) Jaguar (Panthera onca) feeding ecology: distribution of predator and prey through time and space. *Journal of Zoology*, **270**, 25-30.
Welch, H.E., Crawford, R.E. & Hop, H. (1993) Occurrence of Arctic cod (Boreogadus saida) schools and their vulnerability to predation in the Canadian High Arctic. *Arctic*, 331-339.
Węsławski, J.M., Kwaśniewski, S., Stempniewicz, L. & Błachowiak-Samołyk, K. (2006) Biodiversity and energy transfer to top trophic levels in two contrasting Arctic fjords. *Polish Polar Research*, **27**, 259-278.
Williams, J.B., Lenain, D., Ostrowski, S., Tieleman, B.I. & Seddon, P.J. (2002) Energy expenditure and water flux of Rüppell'sfoxes in Saudi Arabia. *Physiological and Biochemical Zoology*, **75**, 479-488.
Williamson, N.J. & Traynor, J.J. (1984) In situ target-strength estimation of Pacific whiting (*Merluccius productus*) using a dual-beam transducer. *Journal du Conseil*, **41**, 285-292.
Wilson, T.M. & Carey, A.B. (1996) Observations of Weasels in Second-Growth Douglas-Fir Forests in the Puget Trough, Washington. *Northwestern Naturalist*, **77**, 35-39.
Wolcott, T.G. (1978) Ecological rôle of ghost crabs, *Ocypode quadrata* (Fabricius) on an ocean beach: Scavengers or predators? *Journal of Experimental Marine Biology and Ecology*, **31**, 67-82.
Wolt, R.C., Gelwick, F.P., Weltz, F. & Davis, R.W. (2012) Foraging behavior and prey of sea otters in a soft-and mixed-sediment benthos in Alaska. *Mammalian Biology-Zeitschrift für Säugetierkunde*,
Womble, J.N. & Sigler, M.F. (2006) Seasonal availability of abundant, energy-rich prey influences the abundance and diet of a marine predator, the Steller sea lion Eumetopias jubatus. *Marine Ecology Progress Series*, **325**, 281-293.
Wright, R.M. (1990) Aspects of the ecology of bream, Abramis brama (L.), in a gravel pit lake and the effects of reducing the population density. *Journal of Fish Biology*, **37**, 629-634.
Xavier, J., Rodhouse, P., Purves, M., Daw, T., Arata, J. & Pilling, G. (2002) Distribution of cephalopods recorded in the diet of the Patagonian toothfish (*Dissostichus eleginoides*) around South Georgia. *Polar Biology*, **25**, 323-330.
Zerbini, A.N. & Santos, M. (1997) First record of the pygmy killer whale *Feresa attenuata* (Gray, 1874) for the Brazilian coast. *Aquatic Mammals*, **23**, 105-110.
